# Physiologically based pharmacokinetic modelling of atomoxetine with regard to *CYP2D6* genotypes

**DOI:** 10.1038/s41598-018-30841-8

**Published:** 2018-08-17

**Authors:** Se-Hyung Kim, Ji-Young Byeon, Young-Hoon Kim, Choong-Min Lee, Yun Jeong Lee, Choon-Gon Jang, Seok-Yong Lee

**Affiliations:** 1 School of Pharmacy, Sungkyunkwan University, Suwon, 16419 Republic of Korea; 2 College of Pharmacy, Dankook University, Cheonan, 31116 Republic of Korea

## Abstract

Atomoxetine is a norepinephrine reuptake inhibitor indicated in the treatment of attention-deficit/hyperactivity disorder. It is primarily metabolized by CYP2D6 to its equipotent metabolite, 4-hydroxyatomoxetine, which promptly undergoes further glucuronidation to an inactive 4-HAT-O-glucuronide. Clinical trials have shown that decreased CYP2D6 activity leads to substantially elevated atomoxetine exposure and increase in adverse reactions. The aim of this study was to to develop a pharmacologically based pharmacokinetic (PBPK) model of atomoxetine in different *CYP2D6* genotypes. A single 20 mg dose of atomoxetine was given to 19 healthy Korean individuals with CYP2D6*wt/*wt (*wt = *1 or *2) or CYP2D6*10/*10 genotype. Based on the results of this pharmacokinetic study, a PBPK model for CYP2D6*wt/*wt individuals was developed. This model was scaled to those with CYP2D6*10/*10 genotype, as well as *CYP2D6* poor metabolisers. We validated this model by comparing the predicted pharmacokinetic parameters with diverse results from the literature. The presented PBPK model describes the pharmacokinetics after single and repeated oral atomoxetine doses with regard to *CYP2D6* genotype and phenotype. This model could be utilized for identification of appropriate dosages of atomoxetine in patients with reduced CYP2D6 activity to minimize the adverse events, and to enable personalised medicine.

## Introduction

Atomoxetine is the first non-stimulant medication approved for the treatment of attention-deficit/hyperactivity disorder (ADHD) in children, adolescents, and adults. It has a potent and selective inhibitory effect on norepinephrine reuptake^1,2^. Guidelines from several organizations, including the American Academy of Pediatrics and the National Institute for Health and Care Excellence in the United Kingdom, recommend atomoxetine for the treatment of ADHD in patients of all ages.

One *in vitro* study found that the main metabolic pathway of atomoxetine is its oxidation to 4-hydroxyatomoxetine (4-HAT) by CYP2D6. To a minor degree, CYP2C19 is responsible for the biotransformation of atomoxetine to N-desmethylatomoxetine (N-DAT)^3^. 4-HAT is equipotent to atomoxetine, but promptly undergoes further glucuronidation to 4-HAT-O-glucuronide and therefore circulates in the plasma at very low concentrations^4^. The same study also indicated the effect of the CYP2D6 phenotype on the pharmacokinetics of atomoxetine. CYP2D6 is a highly polymorphic enzyme with more than 113 known variants (https://www.pharmvar.org/gene/CYP2D6). Among others, CYP2D6*3, *4, *5, and *6 are defective alleles that lack CYP2D6 enzyme activity. These alleles occur in ~20% of Caucasians. Approximately 7% of Caucasians are known to be poor metabolizers (PMs) due to presence of two non-functional alleles^5,6^. Oral administration of atomoxetine resulted in prolonged half-life (t_1/2_) and 10-fold lower clearance (CL/F) in PMs compared to extensive metabolizers (EMs) with normal enzyme activity. These pharmacologic changes led to a 5.7-fold higher maximum plasma concentration (C_max_) of atomoxetine and 7.8-fold greater area under the curve (AUC) in PMs compared to that in EMs^4^. Interestingly, the C_max_ of the equipotent 4-HAT was only ~1.3% of that of atomoxetine^4^. Therefore, the pharmacodynamic effects of atomoxetine are primarily due to the parent drug, and the contribution of the metabolite is exiguous. Higher exposure to atomoxetine is not only correlated with its efficacy, but also with higher risk of adverse events, including tachycardia and elevated diastolic blood pressure^7^. The most common nonspecific adverse reactions of atomoxetine in CYP2D6 PMs include dry mouth, depression, tremor and insomnia^8^. Therefore, these patients are recommended to take lower doses of atomoxetine than EMs. In East Asia, the frequency of nonfunctional alleles is low. The most frequent variant in East Asians including Korean, Chinese, and Japanese populations is CYP2D6*10 (45%, 55%, and 38%, respectively)^9–11^. This allele is known to have decreased enzyme activity both *in vitro* and *in vivo*. Previous studies have demonstrated decreased biotransformation of atomoxetine in Chinese and Japanese patients with the CYP2D6*10/*10 genotype^12,13^. We also verified a significant influence of the CYP2D6*10 allele on the pharmacokinetic parameters of atomoxetine in a study with a larger sample size^14^. In that study, the C_max_ and AUC from time 0 to infinity (AUC_inf_) of the CYP2D6*10/*10 genotype group were 1.7-fold and 3.4-fold higher, respectively, than those of EMs with the CYP2D6*wt/*wt (*wt = *1 or *2) genotype (*P* < 0.001 for both). The t_1/2_ and CL/F were also significantly different between the two groups (*P* < 0.001 for both). However, the increase in atomoxetine exposure in the CYP2D6*10/*10 group was not to the extent observed in the *CYP2D6* PMs. Nevertheless, the risk of a higher incidence of concentration-related adverse events cannot be excluded in the CYP2D6*10/*10 group. Physiologically based pharmacokinetic (PBPK) modelling is a mechanistic approach to simulate the pharmacokinetics of xenobiotics based on their specific properties and mammalian physiology. A PBPK model is advantageous compared to conventional models because it considers individual anatomical and physiological parameters, including enzyme expression, and physicochemical compound data^15^. Therefore, PBPK modelling can be used to individualise therapies for special patient groups (such as children and the elderly) and to predict drug-drug interactions^16–18^. A PBPK model can also be useful to predict the effects of genetic polymorphism on the pharmacokinetics of xenobiotics^19^. In this study, we aimed to develop a PBPK model to predict the pharmacokinetics of atomoxetine in the different *CYP2D6* genotype groups. This model was intended to demonstrate the possibility of simulating the pharmacokinetics of atomoxetine, taking into account the factors that affect it, including demographic characteristics, and *CYP2D6* genotype^20^.

## Results

### Pharmacokinetic study

**Figure 1:**
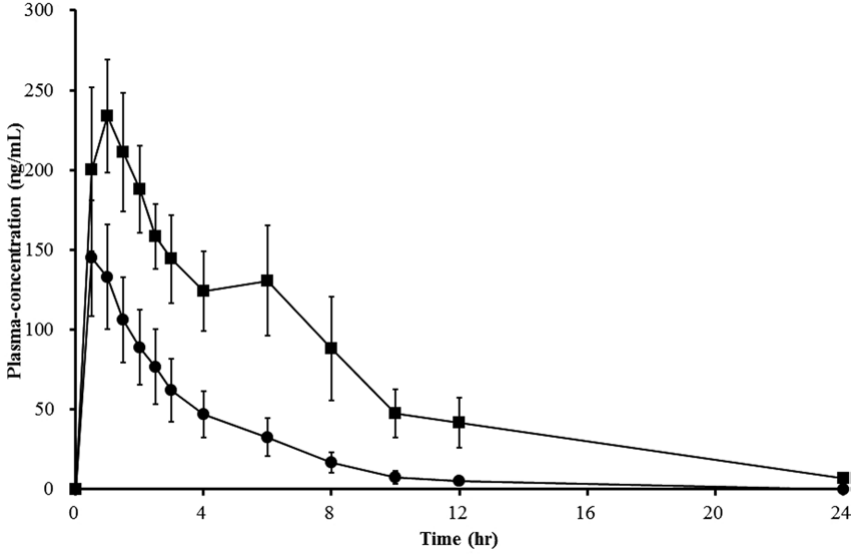
Plasma concentration-time profiles of atomoxetine in CYP2D6*wt/*wt (circles, n = 11) and CYP2D6*10/*10 (squares, n = 8) genotype groups after oral administration of 20 mg atomoxetine. Values represent mean ± SD.

The major alleles found in the 399 Korean subjects were CYP2D6*1 and *2 with normal enzyme activity and *10 with decreased enzyme activity (34.3%, 13.4%, and 47.3%, respectively). The frequency of the nonfunctional allele CYP2D6*5 was 3.5%. Fourteen different genotypes were detected. The frequency of CYP2D6*wt/*wt, heterozygous CYP2D6*10, and homozygous CYP2D6*10 carriers were 23.3%, 45.1%, and 22.1%, respectively. There was only one PM with two defective alleles, while 15 subjects had the CYP2D6*5*/10 genotype. Among 19 healthy subjects enrolled in the pharmacokinetic study, 11 subjects had the CYP2D6*wt/*wt genotype (age range 19–25 years, BMI 18–26 kg/m^2^, weight 49–73 kg) and 8 subjects had the CYP2D6*10/*10 genotype (age range 19–25 years, BMI 18–23 kg/m^2^, weight 52–72 kg). The demographic characteristics were not significantly different between the groups, and all participants completed the study. There were no reported adverse events during and up to 10 days after the study completion. The plasma concentration-time curves and pharmacokinetic parameters of atomoxetine are summarized in Fig. 1 and Table 1. The C_max_, AUC_0–24_, AUC_inf_, t_1/2_, and CL/F were all significantly different between those with CYP2D6*wt/*wt and CYP2D6*10/*10 genotypes (*P* value < 0.0001 for all). Compared to individuals with CYP2D6*wt/*wt, those with CYP2D6*10/*10 had 1.5-fold higher C_max_, 3.1-fold higher AUC_0–24_ and AUC_inf_, and 2.0-fold higher half-life. Oral clearance was 3.0-fold lower in the group with homozygous CYP2D6*10 than in those with the wildtype alleles. The t_max_ was also significantly different between these groups (*P* value = 0.02).

**Table 1:** Pharmacokinetics of atomoxetine in different *CYP2D6* genotype groups after 20 mg oral dose of atomoxetine.

Parameter	CYP2D6*wt/*wt	CYP2D6*10/*10	*P* value
AUC_0–24_ [ng·hr/mL]	503.74 ± 122.60	1587.01 ± 341.03	<0.0001
AUC_0-∞_ [ng·hr/mL]	521.09 ± 130.88	1635.71 ± 362.15	<0.0001
C_max_ [ng/mL]	159.73 ± 24.03	244.63 ± 36.54	<0.0001
CL/F [L/hr/kg]	0.64 ± 0.13	0.21 ± 0.05	<0.0001
t_max_ [hr]	0.50 (0.50–1.00)	1.00 (0.50–1.00)	0.02
t_1/2_ [hr]	2.24 ± 0.28	4.68 ± 0.55	<0.0001

Data are expressed as mean ± SD, except for t_max_, which is expressed as median (range).

### Development of a PBPK model

**Figure 2:**
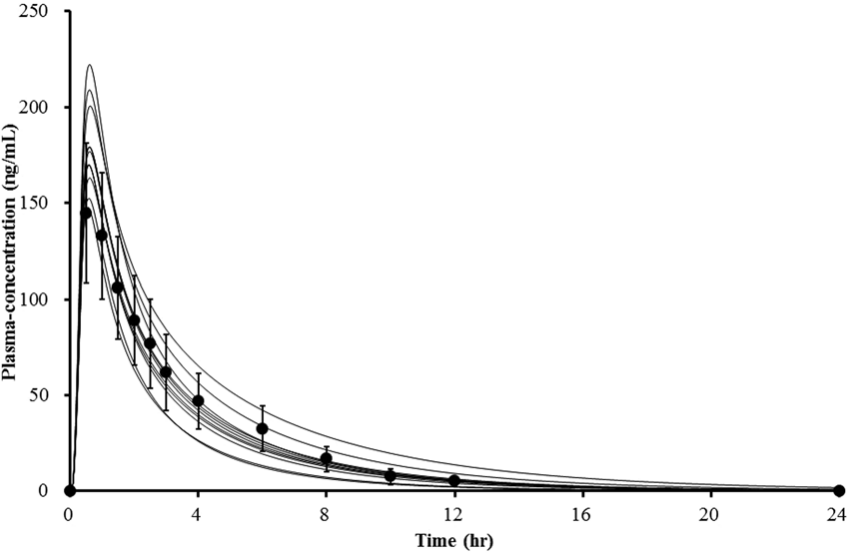
Comparison of simulated (lines) and observed (circles) plasma concentration-time profiles of atomoxetine of CYP2D6*wt/*wt individuals (n = 11). Experimented values represent mean ± SD.

**Figure 3:**
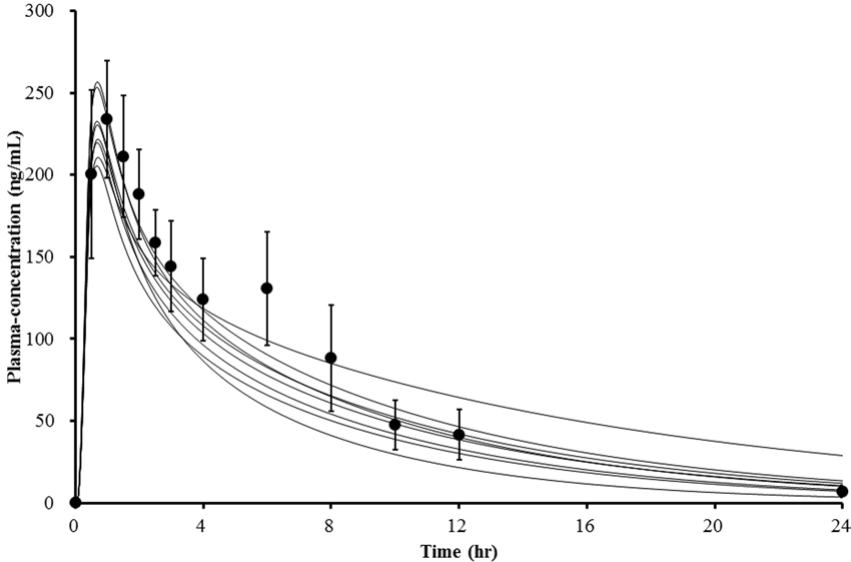
Comparison of simulated (lines) and observed (circles) plasma concentration-time profiles of atomoxetine of CYP2D6*10/*10 individuals (n = 8). Experimented values represent mean ± SD.

**Figure 4:**
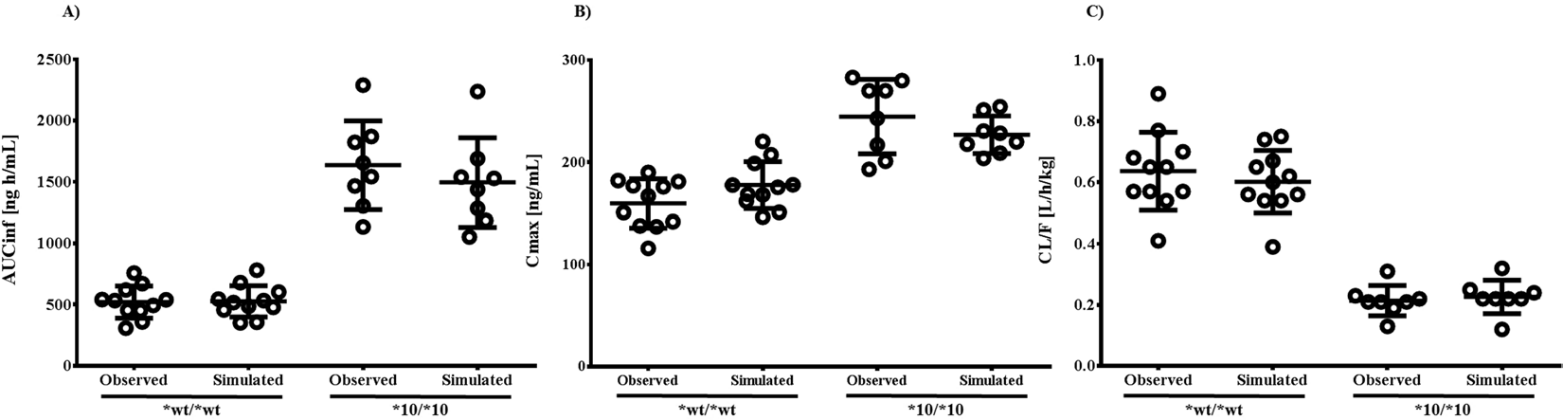
Observed and simulated **(a)** AUC_inf_, **(b)** C_max_, and **(c)** CL/F values after oral administration of 20 mg atomoxetine in relation to *CYP2D6* genotype. Individual values are presented as circles, mean ± SD as lines.

Raw data from the pharmacokinetic study were used to develop the PBPK model. Values for physicochemical and pharmacokinetic (absorption, distribution, metabolism, excretion) parameters, both of which were used in the PBPK model, are listed in Table 2. Optimal value for absorption and distribution permeability was calculated by PK-Sim^®^ based on physicochemical data and plasma concentration profile of atomoxetine. The procedures on which the calculation is based on, are described by Thelen *et al*. and Kawai *et al*.^21–23^. Initially, all of the data were entered as provided in the literature. Specific renal clearance was calculated by the software from plasma clearance as input value. For the paramerisation of clearance, the Levenberg-Marquardt algorithms in the Parameter Identification tool of PK-Sim^®^ were used. The plama clearance values were first inputted individually in order to obtain similar total plasma clearance results to those observed in our pharmacokinetic study, and for the final model, the mean value was used. According to the Pharmaceuticals and Medical Devices Agency in Japan, the dissolution rate of atomoxetine capsules over 30 minutes, which was determined using the paddle method (described in the Japan Pharmacopoeia), was 85% or more. Therefore, 30 minutes was set as the dissolution time^24^. The first simulation showed a slightly lower plasma concentration-time profile than was previously observed. We calculated the organ-plasma partition coefficient based on the method developed by Berezhkovskiy, which is also available in PK-Sim^®^^25^. This method calculates the organ-plasma coefficients using the volume fractions of the aqueous and organic subcompartments of the respective organ and plasma and peripheral drug eliminations are also considered. A sensitivity analysis for the parameters logP, fraction unbound, pKa, plasma clearance, amongst others, was also performed with the Sensitivity Analysis tool provided by the PK-Sim^®^ software. The sensitivity was determined as the mean of several sensitivities based on different relative variations, which were defined by multiplication of the value used for the simulation with variation factors. Both C_max_ and AUC of atomoxetine were identified to be significantly sensitive to logP. After performing the parameter identification as described above, it was adjusted appropriately (logP = 3.8 instead of 3.9). The received curves reflected the observed data better after the adjustment, but they were shifted to the right. This was corrected by reducing the dissolution time to 15 minutes. Finally, the PBPK simulation data adequately fit the experimental data (Fig. 2). The visual comparison of the simulated and observed plasma concentration profiles demonstrated that the shape of the curves were similar. The simulated pharmacokinetic parameters AUC, C_max_ and CL were also in accordance with the experimental results in Table 1. The absorption and distribution were in agreement with the literature. Atomoxetine is known to be well absorbed and the volume of distribution following oral administration is 2.33 L/kg^4^. In our simulation, the oral bioavailability was 96% and the volume of distribution at steady state (V_ss_) was 2.18 L/kg. Apparent volume of distribution V_d_, which is calculated from the plasma curve according to V_d_ = CL/λ, where λ is the terminal elimination rate, was 3.21 L/kg. In the next step, the achieved model was scaled to CYP2D6*10/*10 individuals. In addition to the individual demographic data and plasma clearance value, we modified the *in vitro* metabolic rate of CYP2D6 in the presence of liver microsomes. The value was reduced by around 90% of CYP2D6*1 activity as reported previously^26^. The modified parameters used in the simulation of this genotype group are specified in Table 3. The plasma concentration-time profiles for CYP2D6*10/*10 are shown in Fig. 3. The observed and simulated pharmacokinetic parameters of CYP2D6*wt/*wt and *10/*10 are compared in Fig. 4.

**Table 2:** Atomoxetine parameters used for PBPK development in the CYP2D6*wt/*wt genotype group.

Parameter	Reference value	Input value	Reference/Comment
**Basic Physicochemistry**			
Molecular weight	255.35 g/mol	255.35 g/mol	PubChem
Lipophilicity (logP)	3.9	3.8	PubChem
Fraction unbound	1.2–1.4%	1.3%	Chalon *et al*.^31^
pK_a_	9.23	9.23	MSDS Eli Lilly and Co.
Solubility	27.8 mg/mL	27.8 mg/mL	Sauer *et al*.^20^
**Absorption**			
Specific intestinal permeability		1.48E^−3^ cm/min	Calculated by PK-Sim^®^
**Distribution**			
Specific organ permeability		0.66 cm/min	Calculated by PK-Sim^®^
**Metabolism**			
Metabolic rate of CYP2D6	50 μL/min/mg	50 μL/min/mg	Ring *et al*.^3^
Metabolic rate of CYP2C19	0.75 μL/min/mg	0.75 μL/min/mg	Ring *et al*.^3^
Content of CYP2D6 in liver microsome		10 pmol/mg	PK-Sim^®^ default value
Content of CYP2C19 in liver microsome		19 pmol/mg	PK-Sim^®^ default value
**Excretion**			
Plasma clearance		0.39 L/h/kg	Calculated by PK-Sim^®^
**Formulation**			
80% dissolution time	<30 min	15 min	Lilly^24^

**Table 3:** Modified atomoxetine parameters used in PBPK development for the CYP2D6*10/*10 genotype group and for CYP2D6 extensive and poor metabolisers.

Parameter	Reference value	Input value	Reference/Comment
**(A)** CYP2D6*10/*10			
Metabolic rate of CYP2D6		5 μL/min/mg	Shen *et al*.^26^
Metabolic rate of CYP2C19	0.75 μL/min/mg	0.75 μL/min/mg	Ring *et al*.^3^
Plasma clearance		0.17 L/hr/kg	Calculated by PK-Sim
**(B) CYP2D6 EM**			
Metabolic rate of CYP2D6	50 μL/min/mg	50 μL/min/mg	Ring *et al*.^3^
Metabolic rate of CYP2C19	0.75 μL/min/mg	0.75 μL/min/mg	Ring *et al*.^3^
Plasma clearance		0.25 L/hr/kg	Calculated by PK-Sim
**(C) CYP2D6 PM**			
Metabolic rate of CYP2D6	0.1 μL/min/mg	0.1 μL/min/mg	Ring *et al*.^3^
Metabolic rate of CYP2C19	0.86 μL/min/mg	0.86 μL/min/mg	Ring *et al*.^3^
Plasma clearance		0.030 L/h/kg	Calculated by PK-Sim

### Validation of the PBPK model

From previous published reports, human clinical PK data for atomoxetine in genotype- or phenotype-charaterised subjects were collected and used to validate the developed PBPK model of atomoxetine. The pharmacokinetics were simulated with the created populations (CYP2D6*10/*10, CYP2D6 EMs, PMs) and the adapted dosage regimens from each study. For the simulations of each genotype and phenotype group, only the *in vitro* metabolic rate was adjusted according to *CYP2D6* genotype (Table 3). For the prediction of CYP2D6 EM, plasma clearance of 0.25 L/h/kg was used, which is slightly lower than that in our simulation of CYP2D6*wt/*wt, because the CYP2D6 EM group in the trials incorporated not only CYP2D6*wt/*wt genotype but also those with one decreased or non-functional allele. Besides, values of 0.17 L/h/kg for CYP2D6*10/*10 and 0.030 L/h/kg for PM simulations were inputted to obtain similar total plasma clearances to those reported in each literature. The demographic characteristics of the populations and all predicted C_max_, AUC values with prediction errors were listed with the observed values for comparison in Table 4. All the prediction errors of the simulations were within two-fold error range.

**Table 4:** Demographic characteristics (range of age, BMI, weight) of the population and observed and simulated mean C_max_, AUC and prediction error (PE) of atomoxetine after (A) single administration and (B) at steady state after multiple oral administrations in relation to *CYP2D6* genotype and phenotype.

Reference [Dose] Ethnity	CYP2D6 EM				CYP2D6*10/*10 or PM			Parameter	CYP2D6 EM			CYP2D6*10/*10 or PM		
	Age	BMI	Weight		Age	BMI	Weight		Observed	Simulated	PE (%)	Observed	Simulated	PE (%)
**(A) Single administration**														
This study [20 mg] Korean	19–25 yr	18–26 kg/m^2^	49–73 kg		19–25 yr	18–23 kg/m^2^	52–72 kg	AUC_inf_ [ng·hr/mL]	521.1	513.1	−1.5	1635.7^a^	1443.9	−11.7
								C_max_ [ng/mL]	159.7	177.5	11.1	244.6^a^	227.5	−7.0
Brown *et al*^33^. [10–40 mg] Multi populations	10–18 yr	16–38 kg/m^2^	30–111 kg		13–17 yr	20–32 kg/m^2^	57–102 kg	AUC_inf_ [μM·hr/L]	3.5	3.2	−8.6	49.6	65.9	32.8
								C_max_ [μM/L]	0.7	1.0	42.8	2.5	1.7	−32.0
Matsui *et al*^13^. [10 mg] Japanese	20–31 yr	18–26 kg/m^2^	50–83 kg		20–31 yr	18–26 kg/m^2^	50–83 kg	AUC_inf_ [ng·hr/mL]	331	372.2	12.4	727^a^	698.53	−3.9
								C_max_ [ng/mL]	86.5	85.7	−0.9	125.1^a^	98.69	−21.1
Chalon *et al*^31^. [20 mg] Caucasian & Asian	34–62 yr	22–31 kg/m^2^	60–100 kg		Not incorporated in the trial			AUC_inf_ [ng·hr/mL]	692	575	−16.9			
								C_max_ [ng/mL]	142.2	153	7.6			
Cui *et al*^12^. [40 mg] Chinese	20–39 yr	21–24 kg/m^2^	53–72 kg		20–39 yr	21–24 kg/m^2^	53–72 kg	AUC_inf_ [ng·hr/mL]	2242	1879.3	−16.2	4962^a^	3264.3	−34.2
								C_max_ [ng/mL]	360	357.4	−0.7	530^a^	408.7	−22.9
Matsui *et al*^13^. [120 mg] Japanese	20–31 yr	18–26 kg/m^2^	50–83 kg		20–31 yr	18–26 kg/m^2^	50–83 kg	AUC_inf_ [ng·hr/mL]	4690	4466.6	−4.82	9830^a^	8382.4	−14.7
								C_max_ [ng/mL]	841.3	1028.4	22.2	1270.8^a^	1184.5	−6.8
**(B) Multiple administrations**														
Belle *et al*^30^. [20 mg bid] No information	20-49 yr	23–25 kg/m^2^		Not given	Not incorporated in the trial			AUC_t,SS_ [ng·hr/mL]	846	788.1	−6.8			
								C_max,SS_ [ng/mL]	184	183.3	−0.4			
Sauer *et al*^4^. [20 mg bid] No information	38–54 yr	21–25 kg/m^2^		Not given	19–49 yr	22–26 kg/m^2^	Not given	AUC_t,SS_ [ng·hr/mL]	1080	832.9	−22.9	8440.0^b^	7531.0	−10.8
								C_max,SS_ [ng/mL]	159.7	179.2	12.2	914.7^b^	752.4	−17.7
Sauer *et al*^32^. [60 mg bid] Caucasian & African-American	26–55 yr	19–30 kg/m^2^		Not given	25–35 yr	18–25 kg/m^2^	Not given	AUC_t,SS_ [ng·hr/mL]	2690	2331.7	−13.3	24264^b^	23821.6	−1.8
								C_max,SS_ [ng/mL]	590.8	478.7	−19.0	2693.8^b^	2365.7	−12.2
Cui *et al*^12^. [80 mg] Chinese	20–39 yr	20.8–24 kg/m^2^		53–72 kg	20–39 yr	20.8–24 kg/m^2^	53–72 kg	AUC_t,SS_ [ng·hr/mL]	4427	3773.7	−14.8	9693^a^	6586.0	−32.0
								C_max,SS_ [ng/mL]	815	729.3	−10.5	1199^a^	878.0	−26.8

^a^Clinically observed results of CYP2D6*10/*10.

^b^Clinically observed results of CYP2D6 PM.

## Discussion

Atomoxetine is mainly metabolised by CYP2D6 to 4-HAT, and its pharmacokinetics are strongly affected by *CYP2D6* genotype. The C_max_ and AUC were substantially increased in CYP2D6 PMs compared to those in EMs. The half-life was also prolonged by a factor of 3.7, and clearance was decreased by over 90% in PMs compared to EMs^4,20^. The efficacy and adverse events of a drug are both strongly dependent on drug exposure. In clinical trials, including open-label and long-term studies, PMs experienced more adverse events than EMs and subsequently had to discontinue the medication more frequently than those with adequate metabolism (11.2% of PMs vs. 6.3% of EMs)^8^. In contrast, more EMs (than PMs) stopped taking the drug because of lack of efficacy^7^. Therefore, in the United States and European countries, drug labels of atomoxetine recommend different dosage regimens based on CYP2D6 phenotype. In contrast, there is a low rate of CYP2D6 PMs in East Asian countries, and dosing adjustment recommendations are not present on atomoxetine drug labels in Korea and Japan, for instance. Nevertheless, several studies have demonstrated the effect of the CYP2D6*10 allele, which is found mostly in East Asians, on the pharmacokinetics of atomoxetine^12–14^. The parameters of the pharmacokinetic study conducted in this study were considerably different in the two genotype groups, CYP2D6*wt/*wt and CYP2D6*10/*10. The C_max_ and AUC of individuals homozygous for CYP2D6*10 were significantly higher (1.53-fold in C_max_ and 3.14-fold in AUC) than those of the CYP2D6*wt/*wt genotype group (*P* < 0.001 for both). The oral clearance was lower and the half-life was longer for those homozygous for CYP2D6*10 compared to those with the wildtype allele (*P* < 0.001 for both). While the C_max_ and AUC increases in CYP2D6*10/*10 individuals were not to the extent of 5.7-fold and 7.8-fold respective increases observed in CYP2D6 PMs^4^, an increased risk of concentration-related adverse reactions in these individuals cannot be excluded. There were two other reports^12,13^ comparing the pharmacokinetic parameters of atomoxetine in CYP2D6 EM and CYP2D6*10/*10, and their results also revelaled the significant increase in atomoxetine exposure in CYP2D6*10/*10 than in CYP2D6 EM although the differences in C_max_ and AUC between two genotypes their studies were slightly less than those in this study^12,13^.

PBPK models can be used to predict adequate drug dosing in order to avoid excessive difference in drug exposure: for instance, in individuals of different genotypes. Some previous studies have been conducted regarding the PBPK modelling of atomoxetine. Ball *et al*.^27^ reported a PBPK modelling approach to predict the unbound CNS drug concentration in rats during preclinical drug development. As this PBPK modelling was conducted in rats, it has a limited application in humans. Dinh *et al*.^28^ investigated the biotransformation of atomoxetine by CYP2D6 and other CYP isoforms as a preparation for developing a paediatric PBPK model. However, a PBPK model of of atomoxetine in humans was not reported in their study. Recently, after the first submission of our manuscript, Huang *et al*. reported a human PBPK model of atomoxetine^29^. Their PBPK model was developed using datasets obtained from literature, including human clinical pharmacokinetic data. However, their PBPK model failed to predict atomoxetine disposition in 100% of East Asian populations with CYP2D6 EM or CYP2D6*10/*10 genotypes or phenotypes, drug interaction studies, and specific population with renal disease or hepatic impairment. They validated the PBPK model using drug-specific acceptance criteria but did not succeed in predicting atomoxetine disposition in 100% of East Asian populations with CYP2D6 EM or CYP2D6*10/*10 genotypes or phenotypes, even if they validated by applying two-fold error criteria. Since we carried out our own pharmacokinetic studies, and thus all individual raw data was available, it was possible, in contrast to Huang *et al*., to perform individual simulations for model development. Our simulation results are not only consistent with the data from our pharmacokinetic study, but also with the previously published pharmacokinetic studies (Table 4). Among the nearly 400 genotyped individuals recruited for this study, there was only one CYP2D6 PM with the presence of two nonfunctional alleles (i.e. CYP2D6*5/*5), and this individual gave up participation in the study. Because of the low frequency, it was not possible to recruit PM subjects for the pharmacokinetic study. Therefore, we were unable to perform simulations on PM individuals. Instead, population simulations were performed, and the data were compared to that from the literature^4,30–32^. The developed PBPK model of atomoxetine is not coupled with models of its metabolites. Since the equipotent 4-HAT is immediately metabolised to a glucuronide form, its exposure is expected to only exert a minor effect on ADHD therapy. We were able to develop a suitable model in relation to *CYP2D6* genotype after several adjustments. Regardless, there are a few limitations that must be considered. Only few papers have genotyped the subjects on *CYP2D6*, in which would describe the enzyme activity more accurately, whereas the other papers have phenotyped them. Thus, a group of CYP2D6 EMs might include lots of different combinations of *CYP2D6* alleles, including those with decreased enzyme activity. Therefore, the potential variability of a phenotyped group is larger than a genotype group. Nevertheless, simulations with a plasma clearance of 0.25 L/h/kg, which is smaller than the plasma clearance used for CYP2D6*wt/*wt, described the pharmacokinetics of EM patients accurately. Inter-individual variability in pharmacokinetics is not only dependent on pharmacogenetics. As shown in our study, plasma clearance and/or protein binding vary greatly between individuals. Both of these factors can affect the pharmacokinetic characteristics of atomoxetine. Individual clearance was considered in this study. For protein binding, we used the mean value from the literature. Therefore, our model will not necessarily be able to predict the pharmacokinetics of each individual exactly, but by using the mean values for all parameters, the results of all individuals were within the two-fold error range. Thus the model might be helpful to identify the appropriate doses at the start of therapy by simply inputting the demographics of the patient, which is a factor that influences the pharmacokinetics of atomoxetine, with the presumption that the genotype of the patient is known. The presented model can also be further adjusted to predict drug-drug interactions. Belle *et al*. found that the C_max_ and AUC of atomoxetine in EM subjects increased by 3.5-fold and 6.5-fold, respectively, after co-administration with paroxetine, a strong CYP2D6 inhibitor^30^. These increases are similar to those observed in PMs. Therefore, the dose adjustments in PM patients may also be similarly applied to those with concomitant use of potent CYP2D6 inhibitors. Atomoxetine is a drug that is primarily administered to children and adolescents. Therefore, it is important that data are generalisable to this population. The prediction error of the simulation with paediatric patients was larger than for the other simulations, but still within the two-fold error range. But there was only one dataset in the literature which provided pharmacokinetics of paediatric patients in relation to *CYP2D6*^33^. Further simulations are needed for validation. In conclusion, we have developed a PBPK model of atomoxetine, which predicts the plasma concentration-time profiles of CYP2D6 EMs with CYP2D6*wt/*wt genotype and also of those with reduced enzyme activity due to CYP2D6*10 alleles and those with defective alleles. This model appropriately represents the pharmacokinetics of atomoxetine, considering the factors that affect the pharmacokinetics of atomoxetine, including demographic characteristics, and *CYP2D6* genotype^20^ and therefore may serve as a basis for further model development, i.e. for the simulation of the drug’s pharmacokinetics in special patient groups, such as those with hepatic impairment, or for the prediction of the effects of co-administered drugs.

## Methods

### Subjects

A total of 399 healthy Koreans subjects were recruited, and their genomic DNA was extracted from 10 mL whole blood samples using the Wizard^®^ Genomic DNA Purification Kit (Promega, Madison, WI, USA). Isolated DNA was used to determine the *CYP2D6* and *CYP2C19* genotypes. Detection of CYP2D6*2, *5, *10, *XxN and CYP2C19*2, *3, *17 alleles were performed via polymerase chain reaction restriction fragment length polymorphism (PCR-RFLP) or long range PCR using previously described methods^34^. Nineteen healthy Korean subjects with CYP2D6*wt/*wt or CYP2D6*10/*10 genotype were randomly selected for the pharmacokinetic study of atomoxetine. None of them had a CYP2C19 PM (CYP2C19*2/*2, *2/*3, *3/*3) genotype. Subjects were healthy according to medical history, physical examination, and routine laboratory tests (blood chemistry, hematology, and urine analysis). They were not permitted to use any medications, alcohol, or caffeine for 10 days prior to or during the pharmacokinetic study. All subjects provided verbal and written informed consent.

### Study protocol

The study was carried out in accordance with the guidelines of the Declaration of Helsinki, and the research protocol was approved by the Institutional Review Board of Metro Hospital, Anyang, Republic of Korea. After overnight fasting, the subjects were administered a single oral dose of 20 mg atomoxetine (Strattera^®^, Eli Lilly and Co., Seoul, Republic of Korea) with 200 mL of water. Blood samples were collected before treatment and at 0.5, 1, 1.5, 2, 2.5, 3, 4, 6, 8, 10, 12, and 24 hours after treatment. The blood samples were promptly centrifuged after collection. Plasma samples were kept at −70 °C until analysis.

### Determination of plasma concentration

Plasma concentrations of atomoxetine and its metabolites (4-HAT and N-DAT) were determined and validated using liquid chromatography/tandem mass spectrometry following a previously described protocol^35,36^. Briefly, after extraction with methyl t-butyl ether, analytes in human plasma were chromatographically separated using a Luna C_18_ column (2.0 mm × 100 mm, 3 μm, Phenomenex Inc., Torrance, CA, USA) with a 10:90 (v/v) mixture of 10 mM ammonium formate buffer (pH 3.5) and methanol. Analyte quantification was performed with ESI-MS/MS detection in positive ion mode using multiple reaction monitoring (MRM) mode. The calibration curves were linear over the range of 1–750 ng/mL for atomoxetine, 0.05–20 ng/mL for 4-HAT, and 0.1–20 ng/mL for N-DAT.

### Data and statistical analysis

The pharmacokinetic parameters of atomoxetine, 4-HAT, and N-DAT were calculated by performing non-compartmental analysis with BA calc 2007 (Ministry of Food and Drug Safety, Seoul, Republic of Korea). The C_max_ values and time to reach C_max_ (t_max_) were derived from the experimentally measured values. Area under the curve was calculated using the log-linear trapezoidal approximation. AUC_inf_ was extrapolated by adding C_t_/k_e_ to AUC_0-t_, where C_t_ is the last measured plasma concentration, and k_e_ is the elimination rate constant. The elimination rate constant was estimated from the terminal phase using log-linear regression. The half-life was assessed using the formula t_1/2_ = ln _2_/k_e_. The apparent oral clearance was calculated with the following formula: CL/F = dose/AUC_inf_. All data are presented as mean ± standard deviation (SD), except t_max_, which is expressed as median and range. The differences in pharmacokinetic parameters between the two genotype groups were assessed using the Mann-Whitney rank sum test. SigmaPlot^®^ version 12 (Systat Software, Chicago, IL, USA) was used for the statistical analysis. *P* values < 0.05 were considered statistically significant (α = 0.05). The power of the study was at least 80%. The sample size was estimated to be sufficient to detect 100% greater AUC of atomoxetine among the two genotype groups. The sample sizes were calculated with the Power and Sample Size Program, PS (version 3.0.17)^37^.

### PBPK model development workflow

The modelling software PK-Sim^®^ version 7.1.0 (Open Systems Pharmacology Suite) was used to develop this PBPK model. Demographic information on age, weight, and height were collected during the recruitment for the pharmacokinetic study. Compound specific physicochemical data were obtained from the literature and integrated into the simulation. Values describing the absorption, distribution, metabolism and excretion (ADME) were obtained from the literature or calculated *in silico* with PK-Sim^®^. All other parameters and settings were maintained at default values. If necessary, the input parameters were modified in the appropriate dimensions after performing a sensitivity analysis and parameter identification to better fit the simulated and experimented data. Once the PBPK model for the CYP2D6*wt/*wt genotype group was generated, the CYP2D6*10/*10 genotype model was scaled by adjusting the individual biometric data, the metabolic enzyme activity, and the clearance value. Both simulations were validated with the experimental results from diverse studies. The populations (n = 10) were created using PK-Sim^®^. Different ethnic groups were addressed by selecting the ethnicity provided by the software. The demographic characteristics (age, BMI, weight) provided in the literature were set as upper and lower limits of the population. The proportion of female volunteers was defined equally to each study. The software then randomly selected parameters to be included. We included all observed data from the available literature, which included the pharmacokinetics of adult and paediatric CYP2D6 EM, CYP2D6*10/*10 and CYP2D6 PM. The administration protocols were equally adapted. We created populations of poor metabolisers with two defective *CYP2D6* alleles as described above and similarly compared these data. Paediatric CYP2D6 EM and PM were simulated individually, as all demographic characteristics, their *CYP2D6* genotype and administered dosage were known^33^. This model was evaluated by visual comparison and in terms of the prediction error (PE), calculated as PE = (simulated-observed)/observed × 100. A relative error smaller than a two-fold error was accepted, which is widely applied by the pharmaceutical industry in drug discovery and development^38^. If necessary, model parameter was refined during model verification, as recommended by the United States Food and Drug Administration (FDA) guidance.

## Data Availability

The datasets generated and analysed in the current study are available from the corresponding author on reasonable request.
